# The role of cardiac imaging in the post-ISCHEMIA world

**DOI:** 10.1093/bjr/tqaf085

**Published:** 2025-04-24

**Authors:** Julia Niemierko, Ed Nicol, Jonathan R Weir-McCall

**Affiliations:** 2nd Department of Radiology, Medical University of Gdansk, Gdansk, 80-210, Poland; Department of Cardiology, Royal Brompton Hospital, London, SW3 6NP, United Kingdom; Department of Radiology, Royal Brompton Hospital, London, SW3 6NP, United Kingdom; School of Biomedical Engineering and Imaging Sciences, King’s College, London, SE1 7EH, United Kingdom; Department of Radiology, Royal Brompton Hospital, London, SW3 6NP, United Kingdom; School of Biomedical Engineering and Imaging Sciences, King’s College, London, SE1 7EH, United Kingdom

**Keywords:** coronary artery disease, cardiac computed tomography, cardiac magnetic resonance imaging, SPECT, guidelines

## Abstract

The last decade has seen a significant expansion of the evidence supporting the use of non-invasive imaging in the diagnosis of stable chest pain. The most significant of these was the ISCHEMIA trial which redefined the role of anatomical and functional imaging. The current paper examines this trial in the context of the wider literature, to bring together a review of the role of imaging in the patient with stable chest pain.

## Introduction

In the management of heart disease, patients prioritize 3 things: improving their quality of life, functional status, and lifespan, with the latter most heavily weighted.^1^ To achieve these goals, the first step is to ascribe an aetiology to the patient’s symptoms in order to guide management for symptomatic relief and to improve prognosis. The use of imaging in those with stable chest pain is central to this process, with the use of diagnostic tests for coronary artery disease (CAD) diagnosis steadily rising over the last decade. This increase in imaging is particularly pronounced for coronary computed tomographic angiography (CCTA) and cardiac magnetic resonance imaging (CMR), the use of which are growing by 25% and 18% per year, respectively.^2^

Irrespective of the imaging test used, the diagnosis of the presence of CAD is only the first step. In those with confirmed CAD and chest pain, initial management consists of anti-anginals combined with risk factor management and preventative therapies to reduce the risk of disease progression and acute cardiovascular events.^3^ The question then to be answered is whether more aggressive intervention is required in the form of revascularization? While revascularization improves blood flow to the myocardium distal to any obstruction, it also comes with significant risk of harm, as well as potential benefit. Coronary artery bypass grafting (CABG) surgery has a 2% mortality rate at 30 days,^4^ while coronary stents have a 2% complication rate (restenosis, myocardial infarct, or death) per year, every year after their implantation, with no evidence of a plateau.^5^ It is therefore critical to identify those in whom the benefit from revascularization over optimal medical therapy (OMT) is greater than this harm. Based on historical observational data, the threshold at which the benefit-risk trade-off begins to favour revascularization occurs when inducible ischaemia affects ≥12.5% of the myocardium, with the benefit becoming progressively more pronounced the greater the volume of inducible ischaemia beyond this threshold.^6^

It was this hypothetical threshold of benefit for revascularization that the ISCHEMIA (International Study of Comparative Health Effectiveness With Medical and Invasive Approaches) trial sought to investigate.^7^ Spanning 8 years, with 320 recruitment sites across 37 countries, and costing $100 million, the ISCHEMIA trial is one of the most significant randomized control trials in cardiovascular medicine to date. Totally, 5179 patients diagnosed with moderate-severe ischaemia through functional imaging or exercise electrocardiogram (ExECG) and with a confirmed coronary stenosis exceeding 50% by diameter reduction in at least 1 major epicardial coronary artery, but without significant left main (LM) disease, predetermined by CCTA were enrolled. The trial excluded patients with an ejection fraction (EF) less than 35%, class III or IV heart failure symptoms, or unstable angina. The cohort included high-risk patients, with a high prevalence of coronary risk factors: 77% male, 73% with hypertension, and 42% with diabetes. Both study arms exhibited strong adherence to medical therapy, meeting key targets for statins, aspirin, systolic blood pressure (below 140 mm Hg), and smoking cessation.

Overall, there was a 12% incidence of major adverse cardiovascular events (the primary outcome, including cardiovascular death, non-fatal myocardial infarct or cardiac-related hospitalization), with no survival benefit demonstrated for revascularization versus OMT (hazard ratio 0.93 [95% confidence interval 0.80 to 1.08]).^8^ These results were reinforced by the ISCHEMIA-EXTEND longer term follow-up study, which also found no difference in all-cause mortality at 5 years.^9^

## Previous trials

The ISCHEMIA trial is the most recent of many trials seeking to find prognostic benefit for revascularization versus OMT in stable CAD. In 2007, the COURAGE (Clinical Outcomes Utilizing Revascularization and Aggressive Drug Evaluation) trial compared revascularization versus OMT in patients with either a ≥ 70% stenosis in at least 1 proximal epicardial coronary artery with evidence of myocardial ischaemia; or alternatively, ≥80% stenosis in at least 1 coronary artery and classic symptoms.^10^ The primary outcome—a combination of death from any cause and nonfatal myocardial infarction—was the same in both groups (19.0% in the percutaneous coronary intervention [PCI] group versus 18.5% in the OMT group). These results held true at 15 years follow-up.^11^ The Bypass Angioplasty Revascularization Investigation 2 Diabetes (BARI 2D) trial involved the analysis of 2368 patients with both type 2 diabetes and stable ischaemic heart disease (SIHD).^12^ The diagnosis of CAD was confirmed through angiography, which required ≥50% stenosis of a major epicardial coronary artery associated with a positive stress test or ≥70% stenosis of a major epicardial coronary artery in combination with classic angina. Again, there were no significant differences in the rates of death from any cause between the revascularization group and the medical-therapy group. The 5-year survival rate did not differ significantly between the patients in the revascularization group and the patients in the OMT group (88.3% vs 87.8%). Even invasive measures of lesion-specific haemodynamic significance have failed to improve cardiovascular outcomes, with FAME-2 and FUTURE, both finding fractional flow reserve (FFR) guided revascularization to yield equivalent outcomes to medical therapy.^13^^,^^14^

What this series of trials consistently demonstrates is that, if a patient is on OMT, there is no evidence to support revascularization from a survival perspective. There are of course important caveats to this statement. Patients with LM disease (≥50% stenosis), or LM equivalent disease (≥70% ostial stenosis of the left anterior descending artery [LAD] and left circumflex artery [LCx]) were excluded from ISCHEMIA (found in 434 out of 5757 CCTAs). So were those with ischaemic cardiomyopathy (left ventricular ejection fraction [LVEF] <35%) where there is a proven long term survival benefit from CABG.^15^ It is worth noting that all of these exclusion criteria are based on the anatomical suitability of the patient for CABG, rather than the burden of ischaemia.

## Current guidelines (NICE, ESC, ACC)

Multiple societies have produced guidelines for the investigation and management of patients with SIHD. The first step in all of these is to evaluate the pre-test probability of ischemic heart disease (IHD). The ACC/AHA guidelines use a pre-test probability score based on age, sex, and symptoms, with the ESC PTP also including risk factors, while NICE uses the nature of the chest pain and ECG findings to determine if the pain is likely to be anginal in nature.^16^ In all these guidelines, individuals at low risk require no further testing, whereas those with an intermediate to high pretest probability require further investigation.

The downstream imaging recommendations in all guidelines promote non-invasive testing. CCTA is the first-line test in all-patients in the NICE guidelines, while the ESC and ACC/AHA guidelines give it a level 1 recommendation and consider it a preferred test in younger patients with low-intermediate clinical likelihood of significant CAD and when high-quality imaging is likely. This has been backed by multiple randomized control trials showing the safety of a CCTA approach. The DISCHARGE trial demonstrated superior safety of CCTA compared with invasive coronary angiography (ICA), with no impact on cardiovascular events, while the PROMISE trial showed no difference in outcomes between functional imaging and CCTA, and SCOT-HEART showed improved outcomes in the CCTA arm compared to the standard of care arm.^17–19^ Functional testing is recommended in older patients with high clinical likelihood of significant disease, and those with known disease, where revascularization is more likely to be recommended (ESC, ACC/AHA guidelines). ICA is only recommended in patients with a positive CCTA or functional test who have symptoms which persist despite OMT (ESC), and in those with high-risk CAD on CCTA (ACC/AHA). According to NICE guidelines ICA is recommended as a third line test if functional testing is inconclusive.

## What ISCHEMIA means for the current approach to the investigation of stable chest pain

The ISCHEMIA trial was the first extensive randomized controlled trial in SIHD to encompass patients exhibiting moderate to severe ischaemia on stress testing and confirmed anatomical evidence of CAD through anatomic imaging. This ensured false positive functional imaging was mitigated. Given the lack of improvement in survival with revascularization in this well characterized group, the questions to be answered in stable chest pain is reduced to a simple series of questions:

1. Is there coronary artery disease?*—to guide the initiation of preventative therapy*;
2. Is there moderate to severe stenosis that might be the cause of angina?—*to guide symptomatic relief with anti-anginal therapies;*
3. Is there significant LM stem/3 vessel disease?*—to guide revascularization*?

Given that these questions are predominantly those of anatomy, not function, angiography (CCTA or ICA) appears the most logical approach to answer these. A recent meta-analysis of randomized control trials has shown CCTA to have a 70% lower risk of procedural complications than ICA with no increase in risk of myocardial infarcts or cardiovascular mortality.^20^ It has a high accuracy for the detection of significant stenosis and can more accurately detect and quantify plaque than ICA.^17^^,^^21^ It is also preferred by patients, who favour CCTA over SPECT, stress MRI, and ICA, due to its “fast, uncomplicated, noninvasive, and painless nature.”^22^^,^^23^

## Moving from a stenosis based paradigm

Historically, the question of the presence of significant CAD has been defined as the presence of a 50% stenosis by diameter reduction in an epicardial vessel on planar imaging (ICA). Indeed, this is still the case in the ESC and NICE guidelines. It is only in the most recent ACC/AHA guidelines that non-obstructive CAD was recognized as a distinct and important entity.^24^ The SCOT-HEART trial demonstrated the power of the detection and treatment of early stage disease. In this trial, those randomized to CCTA had a 50% lower rate of myocardial infarction and cardiovascular death (the primary outcome) compared with the standard of care arm.^25^ Those in the CCTA arm had significantly higher rates of statin and antiplatelet prescription, with this targeted based on the presence of atherosclerosis, not the traditional cardiovascular risk score.^26^ The importance of plaque detected by CCTA for predicting cardiovascular events is well documented, providing prognostic information both in the presence of, and absence of, significant stenosis.^27–29^ Furthermore, there is growing evidence that, due to the low cardiovascular risk of those without plaque, there is less value from statin therapy.^30–32^ Pericoronary fat attenuation—a marker of inflammation—may also provide incremental risk stratification, even in those with “normal” CCTA.^33^ Observational data suggest plaque and pericoronary inflammation may both help guide decision making in the choice of medical therapeutics.^33^^,^^34^ Prospective clinical trials such as the TRANSFORM trial (NCT06112418) are now underway examining whether these changes in decisions driven by CCTA findings result in improved outcomes.

**Figure 1.**
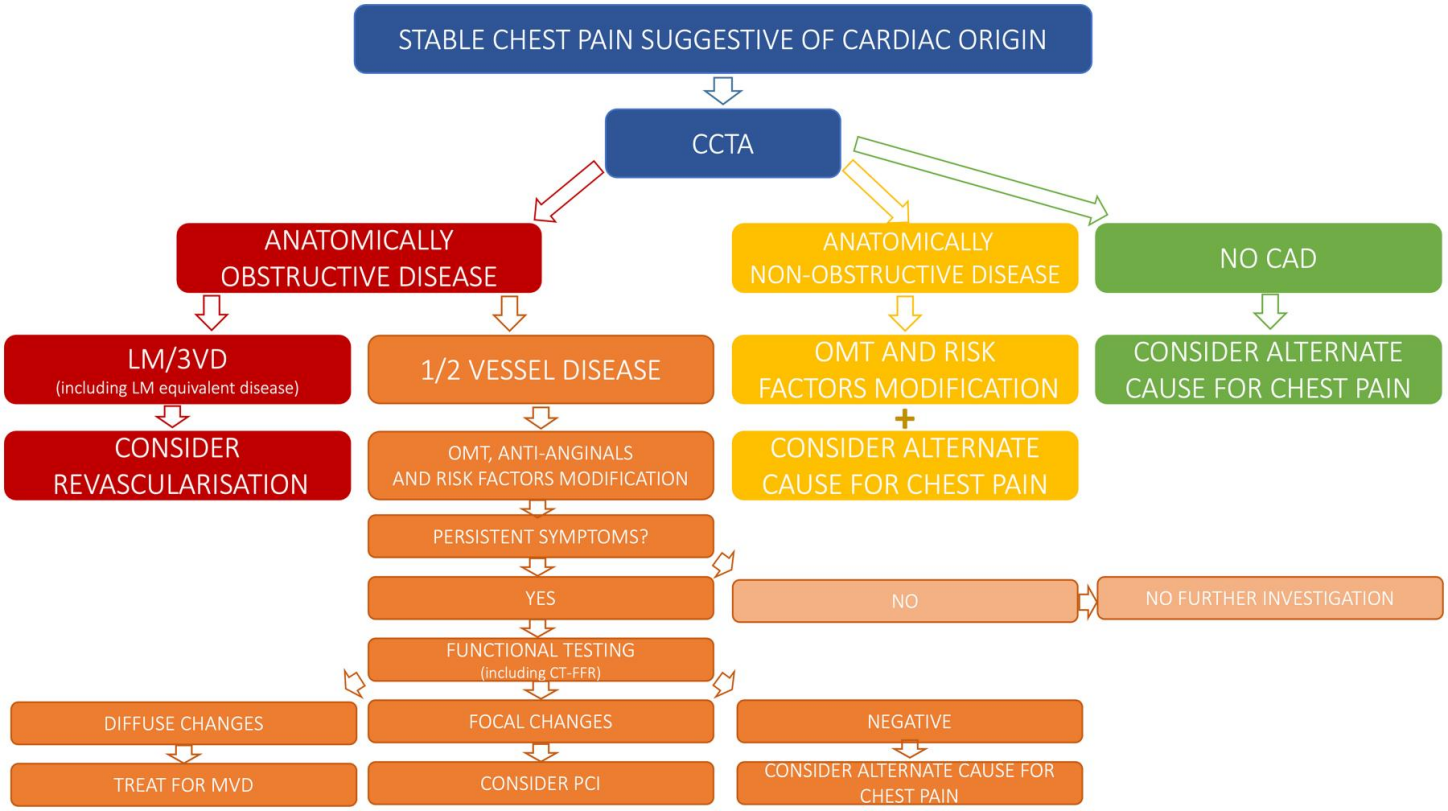
Proposed pathway for assessing patients with stable chest pain suggestive of chronic coronary syndrome. Abbreviations: CAD = coronary artery disease; LM = left main coronary disease; MVD = microvascular dysfunction; OMT = optimal medical therapy; PCI = percutaneous coronary intervention; 3VD = triple vessel coronary artery disease.

Severe CAD was historically viewed as the Achilles heel of CCTA due to the impact of blooming artefact due to coronary calcification and motion artefact in those with irregular motion in AF. However, in a post hoc analysis in the ISCHEMIA trial of the 1728 patients who went on to have an ICA, CCTA showed a high level of agreement with ICA for the absence of LM disease, and for the presence of stenosis >50% in the other coronary arteries, despite the high risk and disease burden of the population.^35^ Moreover, the number of stenosis and severity of stenosis detected on CCTA was accurate in predicting cardiovascular events in this high-risk group, with no additive benefit of ICA.^36^ Given the accuracy of CCTA, and the fact that the decision to revascularize is based on anatomy rather than physiology, the need for ICA as an intermediate step at all is now being actively investigated. Several studies have demonstrated that CABG guided by CCTA (in the absence of ICA) is safe and reliable^37^^,^^38^ (Figure 1).

## The role of revascularization for symptoms relief

Despite the lack of improvement in cardiovascular mortality, a consistent finding across COURAGE, FAME 2 and ISCHEMIA was a more rapid resolution of symptoms in the revascularization group.^10^^,^^39^^,^^40^ Improvements in angina were achieved at initial follow-up in the revascularization group that were not seen in the OMT arm until 3 years, when the symptom burden between the 2 groups equalized. The investigators of the ISCHEMIA trial employed the Seattle Angina Questionnaire to assess angina-related symptoms, function, and quality of life, revealing that patients undergoing revascularization experienced greater improvements in angina-related health status compared to those on the conservative (OMT) strategy.^40^ They also showed that those with more severe symptoms were most likely to gain benefit from revascularization. It is however noteworthy that none of these trials were blinded, nor were they placebo-controlled design. The impact of the placebo effect in PCI has been recently tested in the ORBITA (Objective Randomized Blinded Investigation with optimal medical Therapy of Angioplasty in stable angina) and ORBITA-2 trials. ORBITA demonstrated that in those with maximal medical therapy, there was no difference in symptom improvement between sham revascularization procedure and PCI.^41^ ORBITA-2 in turn showed that, in those with no anti-anginal therapies (other than GTN), PCI resulted in a significant reduction in the frequency and severity of angina.^42^ This effect was particularly pronounced in those who exhibited typical angina.^43^ Thus, a revascularization strategy continues to be evidence-based for patients with SIHD who, despite OMT, experience recurrent or intolerable angina, or those who would prefer to avoid the need for multiple daily medications.

## Improving the determination of the functional significance of an anatomic stenosis

When considering whether a stenosis should be revascularized, it is necessary to determine whether the lesion is flow limiting. Several options are available to determine the functional significance of stenoses, either indirectly with the use of proxy adjuncts to the anatomic test (invasive or CT-based FFR [FFR/FFR-CT]), or dedicated non-invasive functional imaging to measure perfusion or contractile reserve.

### CT-based FFR

**Figure 2.**
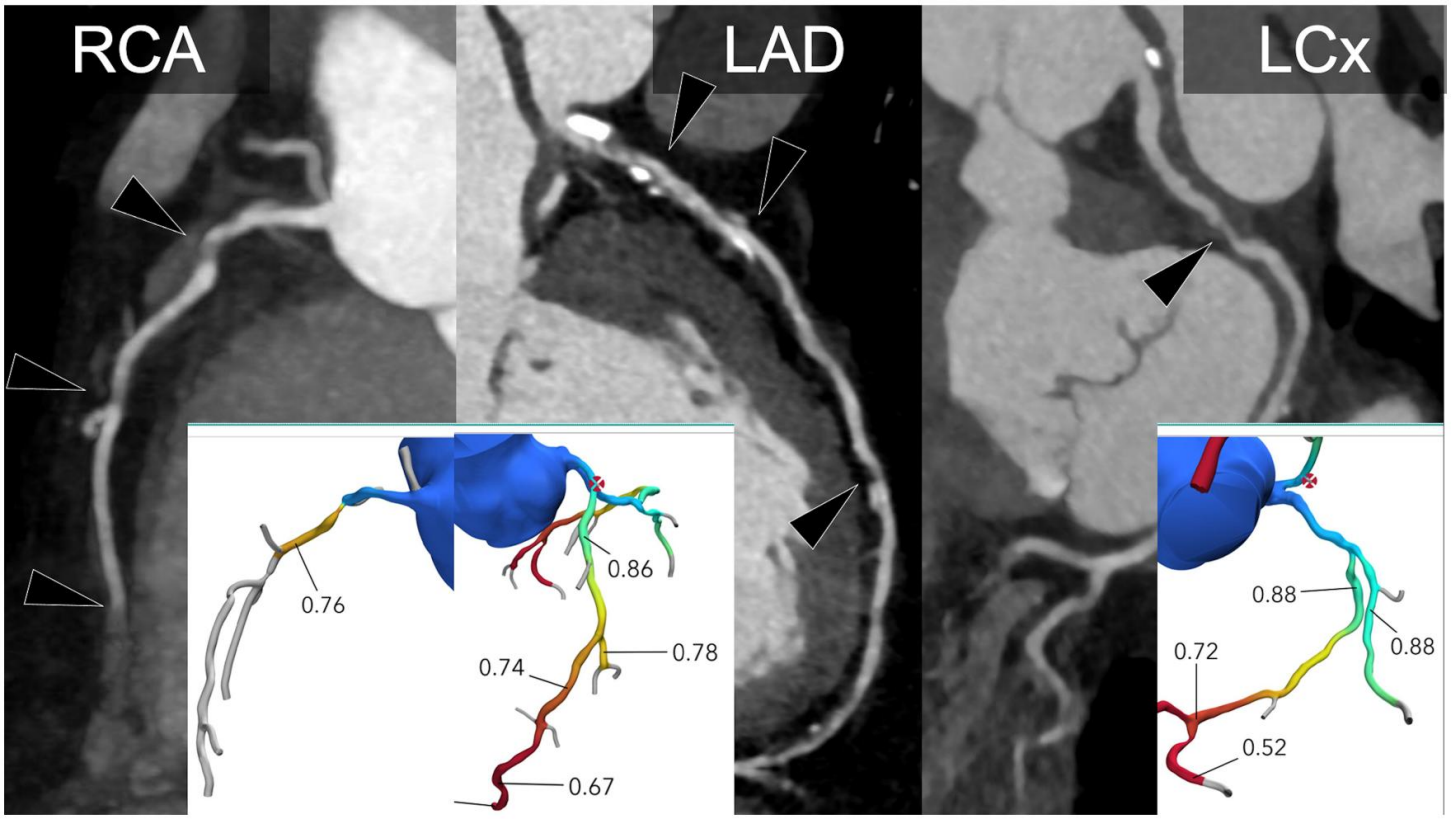
A female patient in her 50s presenting with typical chest pain. Coronary computed tomography angiography (CCTA) reveals multifocal non-calcified plaque causing >70% diameter stenosis in the RCA, multifocal moderate (50%-69%) stenosis in the LAD, and moderate (50%-69%) stenosis in the mid LCx (arrows). Computed tomography-derived fractional flow reserve (CT-FFR) shows the plaque is limiting the blood flow in the RCA, mid LAD, and distal LCx. Findings were confirmed on subsequent ICA, with the patient undergoing coronary artery bypass, with grafts to the LAD, OM1, and left-posterior descending artery. Abbreviations: LAD = left anterior descending artery; LCx = left circumflex artery; RCA = right coronary artery.

Historically, FFR was assessed during ICA. This involved the placement of a pressure guidewire beyond a stenotic lesion, with measurements taken of the mean distal coronary pressure, and compared to the mean aortic pressure, to give a ratio under conditions of maximum hyperemia. FFR quantifies the ratio of maximal (hyperaemic) myocardial blood flow (MBF) downstream of a stenotic lesion to the theoretical normal hyperemic myocardial flow within the same artery.^44^ CCTA has shown high diagnostic sensitivity in identifying coronary stenoses; however, in 1 major study in the early days of CCTA, only 49% of significant stenoses detected by CTCA were found to be flow limiting on invasive FFR.^45^ This was due to both calcification causing blooming artefact and the comparatively limited spatial resolution of CCTA compared with ICA, as well as the discordance between anatomy and physiology. FFR calculated on CTCA datasets (FFR-CT) is a non-invasive diagnostic tool which can be used to assess the functional significance of coronary artery stenosis without an invasive procedure. It demonstrates a high level of correlation with invasive FFR.^46^ The most commonly used method combines the anatomy from the CCTA with computational fluid dynamics to calculate a simulated FFR. However, there are also algorithms which use either generalized microvascular resistance parameters or geometric coronary features to estimate the FFR.^47^ Patients with stable CAD without significant lesion-specific pressure drop on CT-FFR exhibit a low subsequent likelihood of cardiovascular events.^48^ This observation persists even in those with elevated coronary artery calcium scores, although further evidence is required to understand the role of CT-FFR alongside plaque quantitation, particularly with longer term follow-up.^49^ The FORECAST and PRECISE trials compared CCTA±FFR-CT with standard of care for the diagnosis of stable chest pain. Both found FFR-CT reduced the number of subsequent ICAs, with no increase in the risk of MACE and while also cost effective (PRECISE)/cost neutral (FORECAST).^50–52^ A significant criticism of these studies however was that they compared CCTA±FFR-CT with functional testing, with uncertainty as to whether the benefits seen were due to the baseline CCTA or the additional FFR-CT. The TARGET trial examined this question, randomizing 1216 patients with an intermediate (30%-90%) stenosis on CCTA to either FFR-CT or stress test. Those in the FFR-CT arm were less likely to go for an ICA, and of those going for ICA were more likely to have obstructive disease requiring revascularization, than those in the stress test arm.^53^ A key strength of FFR-CT is that it makes use of data already acquired as part of the diagnostic CCTA and requires no additional procedures or hospital visits for the patient. However, FFR-CT requires high-quality CCTA data with minimal motion artefact, and approximately 10% of patients cannot have a FFR-CT analysis of their scan due to sub-optimal image quality seen in clinical practice^54^ (Figure 2).

### Stress imaging

**Figure 3.**
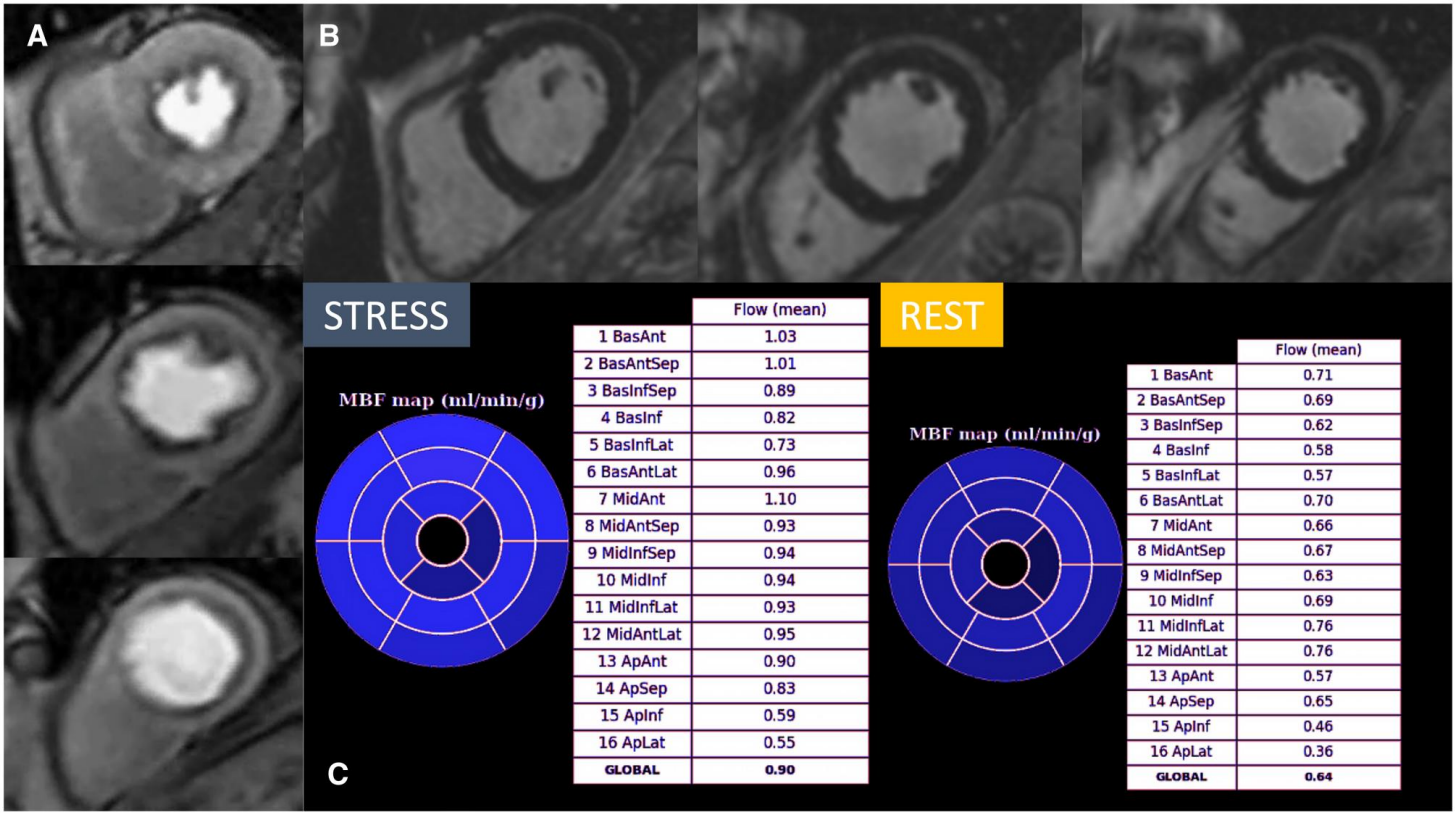
Cardiac MR images of a patient with coronary microvascular dysfunction (MVD): Short-axis stress perfusion images (A) reveal diffuse inducible subendocardial perfusion defects across various vascular territories, whereas late gadolinium enhancement (LGE) images (B) show no visible scar. In some cases, patients with MVD may present without visually apparent subendocardial perfusion defects; however, quantitative perfusion analysis (C) may reveal poor hyperemic blood flow, with a significantly reduced myocardial perfusion reserve (MPR), which can be calculated by dividing stress myocardial blood flow (MBF) by rest MBF.

**Figure 4.**
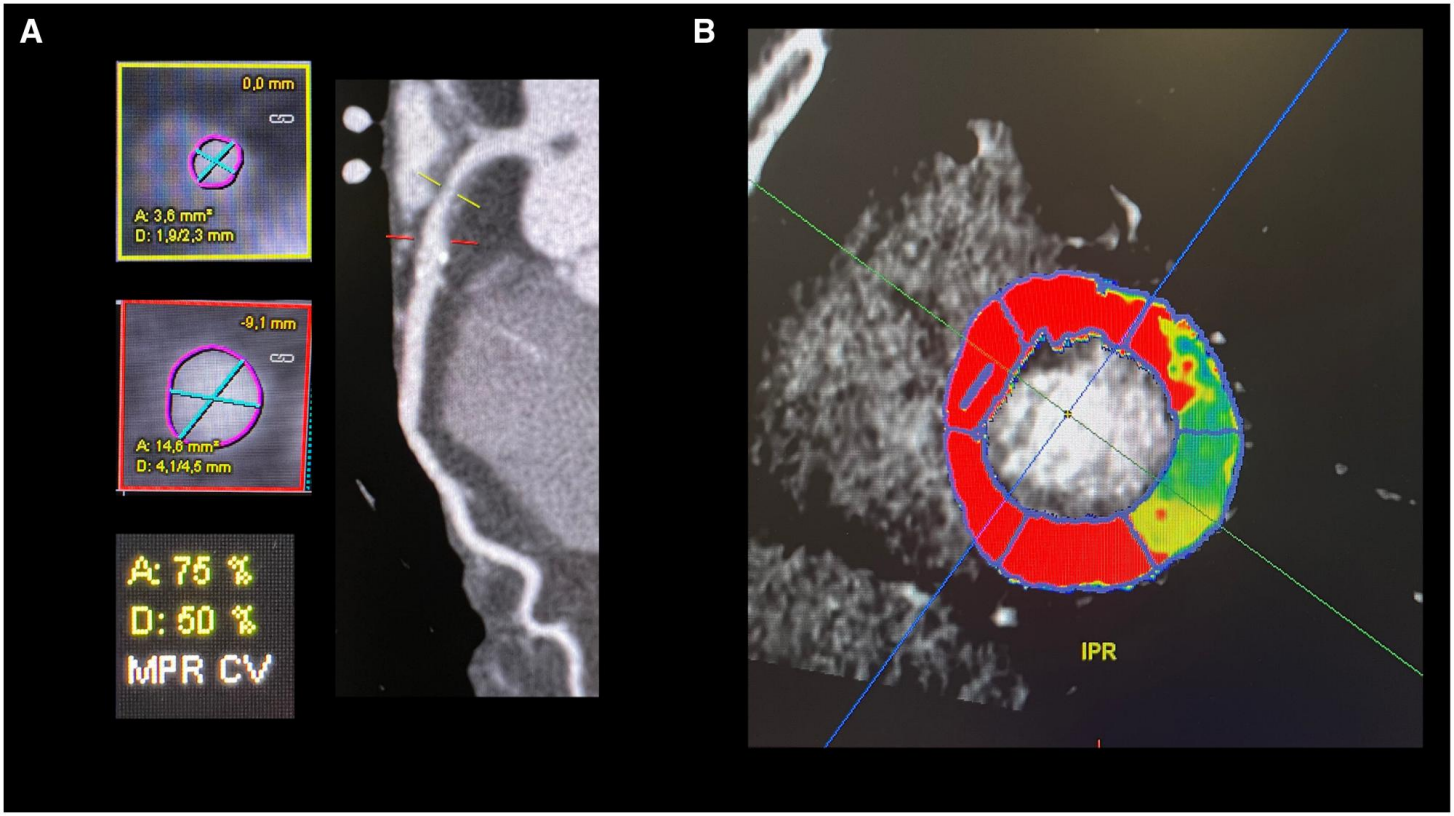
A man in his 70s undergoing investigation for suspected coronary artery disease. (A) Coronary computed tomography angiogram (CCTA) reveals a partially calcified plaque in the proximal segment of the left anterior descending artery (LAD), resulting in approximately 50% diameter stenosis, and 75% area stenosis of the lumen. Subsequently, the patient underwent dynamic computed tomography perfusion imaging, which, as shown in the short-axis plane (B), exhibited impaired perfusion in the corresponding myocardial segments. Abbreviations: MPR CV = multiplanar reformat of coronary vessel.

Stress imaging identifies inducible ischaemia using the surrogates of either stress-induced perfusion defects (SPECT, PET, CT, and perfusion MRI) or inducible wall motion abnormalities (exercise/dobutamine echo and MRI) within a coronary vascular territory that are absent at rest. Both hypoperfusion and regional wall motion abnormalities have a high accuracy for detecting flow limiting disease compared with invasive FFR.^55^ Currently, the presence of inducible ischaemia is predominantly determined by a qualitative visual inspection. This can be supplemented by quantitation of blood flow (MBF and coronary flow reserve [CFR]) using PET, dynamic CT perfusion or dedicated quantitative perfusion sequences in cardiovascular magnetic resonance perfusion. A CFR <2 (the ratio between stress and resting blood flow) indicates the presence of inducible ischaemia, associated with adverse cardiovascular outcomes.^56^ One of the key benefits of CFR is in the detection of 3-vessel disease which can be missed by visual inspection due to homogeneous global perfusion reduction without relative defects and provide improved prediction of future adverse event risk.^57^ Reduced CFR with no visual defects may also result also from microvascular dysfunction, prompting the recommendation of anatomical imaging for differentiation (Figure 3). Anatomical imaging is also important when analysing CT perfusion studies, which although exhibiting good sensitivity for the detection of perfusion deficits have low specificity when analysed independently of CCTA^58^ (Figure 4).

FFR-CT and perfusion imaging each have their own strengths and limitations. As highlighted, FFR-CT requires high-quality imaging and does not provide information on myocardial scar/viability. By comparison, perfusion imaging can identify both inducible ischaemia, impaired myocardial function and, in the case of MRI and PET, myocardial viability. While there are many studies examining the accuracy of FFR-CT,^46^ and myocardial perfusion imaging,^59^ these have almost exclusively been performed in cohorts with chest pain with no information other than risk factors and symptoms. However, in a diagnostic pathway where CCTA is the first-line test, the anatomy is already known. This means those undergoing further testing are likely to have at least an intermediate stenosis in an epicardial vessel. This information significantly alters the pre-test probability, which in turn impacts the post-test likelihood of significance in line with Bayes theorem. A succession of trials examining second-line testing in those with intermediate stenosis (ReASSESS, DAN-NICAD and DAN-NICAD 2) demonstrate the accuracy of both stress testing and FFR-CT are substantially lower than in trials where these tests were first line with no pre-selection.^60–63^ In all these trials, FFR-CT was more sensitive for flow limiting disease, while PET, SPECT, and cardiac MRI were more specific with equivalent overall accuracy. The ultimate choice between these approaches will centre on local test availability, as well as the clinical scenario. In patients with regional wall motion abnormalities on echo, perfusion imaging is useful to examine for myocardial scar and ischaemia. In comparison, FFR-CT can be useful to determine if there is a targetable lesion associated with a focal pressure drop which may be amenable for stenting, or whether the disease is diffuse with this latter phenotype likely gaining less benefit from revascularization.^64^^,^^65^

## Ischaemia with no obstructive coronary artery disease—ISCHAEMIA-CIAO

Ischaemia with no obstructive coronary artery (INOCA) disease typically arises secondary to coronary microvascular dysfunction or vasospasm, and its presence is associated with an elevated risk of cardiovascular events.^66^ In the CorCTCA (Coronary Microvascular Function and CT Coronary Angiography) trial, consecutive patients with no evidence of obstructive CAD on CCTA were invited for invasive endotyping which revealed either microvascular dysfunction or vasospasm in 66% of the patients.^67^ Despite this high prevalence of disease, when patients were randomized to treatment based on the invasive testing versus blinded treatment without disclosing the results of invasive testing, there was no difference in the long-term burden of angina between the 2 groups. A similarly challenging picture was seen in the ISCHEMIA trial. Among participants with core laboratory-confirmed moderate or severe ischaemia in ISCHEMIA, the incidence of INOCA was 13%.^68^ Patients with INOCA were younger, predominantly female, and exhibited fewer atherosclerosis risk factors. This group of patients, who were not included in the main ISCHEMIA trial, were recruited and followed up in the CIAO ISCHEMIA study. This found no relationship between the severity of INOCA (extent of inducible wall motion abnormality) and the severity of angina. Furthermore, 40% of patients’ symptoms resolved spontaneously at 1 year, with no association between the degree of improvement of symptoms and the degree of improvement in the severity of INOCA.^69^ A potential limitation of the study was that it used stress echocardiography rather than quantitative perfusion or invasive measures of vascular function. Given the results of CorCTCA and CIAO ISCHEMIA, routine investigation for INOCA in those with no obstructive CAD on CCTA cannot be recommended. However, further testing may be useful in those with severe or intractable symptoms despite OMT.

## Conclusion

The diagnostic pathway for patients presenting with stable chest pain of suspected coronary origin is undergoing a paradigm shift. ISCHEMIA convincingly showed that conservative management, coupled with evidence-based pharmacologic secondary prevention, is a safe and effective strategy for most patients with stable CAD, who do not have LVEF of <35% or high-risk CAD. This coupled with the evidence in the wider literature suggest that a CCTA first approach enables the identification of patients across the whole spectrum of CAD severity, requiring targeted treatment. A shift in the evaluation of stable IHD, emphasizing the importance of diagnosing coronary atherosclerosis rather than solely focusing on ischaemia, should be the new normal, with functional testing reserved for those with severe or refractory symptoms.
